# Associations of Topics of Discussion on Twitter With Survey Measures of Attitudes, Knowledge, and Behaviors Related to Zika: Probabilistic Study in the United States

**DOI:** 10.2196/publichealth.8186

**Published:** 2018-02-09

**Authors:** Mohsen Farhadloo, Kenneth Winneg, Man-Pui Sally Chan, Kathleen Hall Jamieson, Dolores Albarracin

**Affiliations:** ^1^ University of Illinois at Urbana-Champaign Champaign, IL United States; ^2^ Annenberg Public Policy Center University of Pennsylvania Philadelphia, PA United States

**Keywords:** Zika, Twitter, topic modeling, public policy, public health

## Abstract

**Background:**

Recent outbreaks of Zika virus around the world led to increased discussions about this issue on social media platforms such as Twitter. These discussions may provide useful information about attitudes, knowledge, and behaviors of the population regarding issues that are important for public policy.

**Objective:**

We sought to identify the associations of the topics of discussions on Twitter and survey measures of Zika-related attitudes, knowledge, and behaviors, not solely based upon the volume of such discussions but by analyzing the content of conversations using probabilistic techniques.

**Methods:**

Using probabilistic topic modeling with US county and week as the unit of analysis, we analyzed the content of Twitter online communications to identify topics related to the reported attitudes, knowledge, and behaviors captured in a national representative survey (N=33,193) of the US adult population over 33 weeks.

**Results:**

Our analyses revealed topics related to “congress funding for Zika,” “microcephaly,” “Zika-related travel discussions,” “insect repellent,” “blood transfusion technology,” and “Zika in Miami” were associated with our survey measures of attitudes, knowledge, and behaviors observed over the period of the study.

**Conclusions:**

Our results demonstrated that it is possible to uncover topics of discussions from Twitter communications that are associated with the Zika-related attitudes, knowledge, and behaviors of populations over time. Social media data can be used as a complementary source of information alongside traditional data sources to gauge the patterns of attitudes, knowledge, and behaviors in a population.

## Introduction

Outbreaks of Zika virus in 2016 in various areas of the world [1] led to increased communications about this issue on Twitter and other social media platforms. These communications may provide digital markers of attitudes, behaviors, and knowledge in a population, thus supplying an easily accessible thermometer of variations in psychological responses that are important for public policy. In this paper, our objective was to identify these markers by correlating Twitter data with survey measures of attitudes, behaviors, and knowledge in a representative sample of the US adult population.

Studying attitudes, knowledge, and behaviors has a longstanding theoretical interest. People’s attitudes and knowledge about the world allow them to make behavioral decisions and avoid public health threats throughout the course of their lives. An attitude is regarded as an evaluation of an object as positive or negative [2-3]. For example, an attitude relevant to Zika may entail favoring policies that can reduce infection, such as spraying. These attitudes may be linked to knowledge—factual information about the life cycle of the *Aedes* mosquitos and types of transmission of the Zika virus, for example. Attitudes may be assessed with semantic differential, Likert, or other items tapping evaluations, whereas knowledge measures involve true/false determinations about factual statements about Zika. Attitudes and knowledge as well as behaviors such as repellent use or calls for action on Congress are classic psychological responses to public health information [3]. In decision making and behavior studying, theories of reasoned action [4] and planned behavior [5] specify a limited number of psychological variables that influence a behavior: (1) intention, (2) attitude toward behavior, (3) subjective norms, and (4) perceived behavioral control. Also, there are studies that have investigated the relationships among knowledge, belief, and behavior [6-7] and studies that involve social media along with other psychological variables [8].

Designing a health communication strategy often requires an understanding of how messages may be linked to the attitudes, knowledge, and behaviors of an audience. With the advent of online communication technologies, this understanding can be derived from the analysis of social media data. Twitter, for example, has been used to predict the age, gender, and political orientation [9-13], level of depression [12], and emotions and attitudes [9] of social media users. The work of De Choudhury et al [12] has discovered that those with major depressive disorder have less Twitter activity and higher self-attentional focus and express greater negative emotion, relational and medical concerns, and a greater number of religious thoughts. Also, an analysis of Facebook postings in relation to personality found variations in language with respect to personality, age, and gender [14]. This research suggests that Twitter may be useful in identifying discussion topics in the Zika domain as well.

In this study, we investigated the possibility of discovering topics of discussion on Twitter that are related to the ongoing public health challenge of Zika virus and whether their variations reveal important information regarding changes of Zika-related attitudes, knowledge, and behaviors of populations over time. To discover the topics of discussion, we used probabilistic topic modeling techniques and examined different weighting schemes (binary, term occurrence, and term frequency–inverse document frequency [tfidf]) on the learned models.

## Methods

### Overview

We examined Twitter data to identify topics in the content of the online communications that were about Zika. Employing latent Dirichlet allocation (LDA), we analyzed tweets aggregated based on location information and used the learned models to infer the probability of occurrence of each topic over time using weekly aggregated test tweets. To train the topic models, different weighting schemes (binary, term occurrence, and tfidf) were compared to find the model with the best weighting scheme. We then explored the associations of topics that showed variability over time with weekly aggregated measures of Zika attitudes, knowledge, and behaviors obtained from a survey representing the US adult population. The resulting correlations were used to describe the topics of discussion associated with the psychological measures of our samples.

### Twitter Data

Our Zika corpus was collected from the Twitter network by searching for a set of Zika-related keywords (“Zika,” “dengue,” “yellow fever,” “Zika virus,” “Zika fever,” “flaviviridae,” “brain shrink,” “fetal brain disruption sequence,” “mosquitoes,” “birth defects,” “insect bites,” “mosquito bites,” “insect-borne virus,” “mosquito-borne virus,” “microcephaly,” and “Guillain-Barre syndrome”) using Twitter streaming application programming interface. The resulting dataset contains 3.8 million tweets from February 1, 2016, to August 30, 2016. Using location information, we were able to map about 10% of all the tweets in our corpus into 2695 different US counties. The rest of the tweets (the other 90%) were aggregated using the timestamp information of each tweet, assigning each to weekly documents. We aggregated tweets for the weeks of February 16, 2016, to August 18, 2016, to match the survey data described next.

### Survey Data

The Annenberg Public Policy Center of the University of Pennsylvania designed and carried out a survey of attitudes, knowledge, and behaviors relevant to Zika virus over 33 weeks (N=33,193). Table 1 summarizes the attitude, knowledge, and behavior questions that were asked of the participants each week. Each week, a dual-frame sample was designed to represent the adult US population (including Hawaii and Alaska). A fully replicated, single-stage, random-digit-dialing sample of landline telephone households, along with randomly generated cell phone numbers, was employed. Each weekly wave consisted of 1000 interviews of which at least 600 were obtained from cell phone respondents. Within each landline household, a single respondent (the youngest adult) was selected. Because the interview could take place outside the respondent’s home, cell phone respondents were considered separately from landlines. Surveys were conducted in 5-day intervals, in English and Spanish, typically from Wednesday through Sunday to include both weekdays and weekends.

Each weekly wave was weighted to provide nationally representative and projectable estimates of the adult population 18 years of age and older. The weighting process took into account the disproportionate probability of household and respondent selection due to the number of separate telephone landlines and cell phones answered by respondents and their households, as well as the probability associated with the random selection of an individual household member. Following application of the weights, the sample was post-stratified and balanced by the key demographics of age, race, sex, region, and education. The sample was also weighted to reflect the distribution of phone usage in the general population, meaning the proportion of those who are cell phone only, landline only, and mixed users (see Multimedia Appendix 1 for more details regarding the survey design and data collection method). Our data included 51.61% females, 39.58% college-educated participants (14.88% with some college and 45.54% with high school education or less), 37.21% living in regions at risk for Zika virus, 45.99% aged 18 to 44 years (17.68% ages 45 to 54 years and 36.33% aged 55 years or older), and 5.57% with current/intended pregnancy. The average response rate over the weeks was 7.50%, a figure comparable to that of other national surveys conducted in the United States [15-16].

### Data Analysis

**Figure 1:**
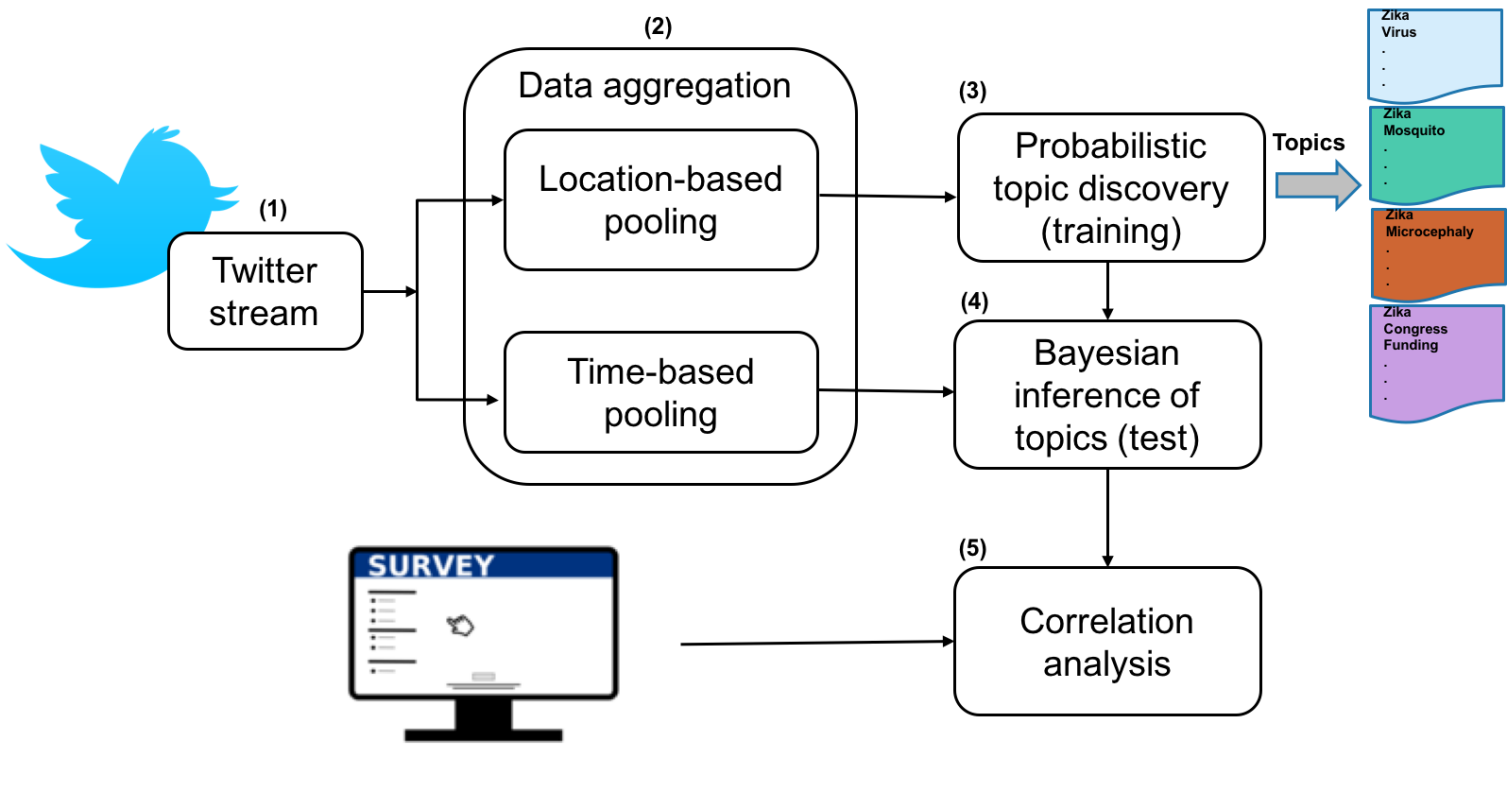
Flow of data processing and analyses.

We analyzed the content of a sample of communications from Twitter (within a 10-month time span) following the process depicted in Figure 1.

We analyzed the sampled tweets using the topic modeling of LDA [17] to uncover topics that users addressed in their communications. This method is a probabilistic way to discover salient patterns (topics) in a collection of text documents; along with its multiple variations, this approach has been used to analyze long news articles, blogs, and scientific papers in various domains [18]. However, optimal applications of LDA to Twitter data deserve further attention because a single tweet is short (140 characters), uses informal language, and contains misspellings, emoticons, acronyms, and nonstandard abbreviations, as well as Twitter names, hashtags, and URLs.

When LDA-based methods are applied directly to posts from microblogging platforms (considering each single tweet as a document), which are usually short and often noisy, these methods result in topics that are uninformative and hard to interpret [19]. To improve the performance of topic modeling of tweets, we incorporated 2 aggregation techniques (Figure 1, phase 2). In order to amass a sufficient number of documents in our training set and generate longer documents, before learning the topic models we created a document for each county in the United States by aggregating all tweets of a county. Not all tweets and Twitter accounts are associated with location information. Typically, 1% of Twitter users have enabled the geocoordinate mobile device service, which tags each tweet with their current geocoordinates [20]. Additionally, some Twitter users have completed the free response location field in their Twitter account profile. For the tweets that contain geolocation coordinates, we found the county corresponding to the coordinates. In addition to using the precise coordinates to locate counties, we geotagged tweets based on the location field when information about a city/state pair or a city name was included. These methods have been described elsewhere [20-21] and were used to generate the training set of tweets. The testing set used in analyses (Figure 1, phase 2) included the tweets that did not have any location information, which comprised 90% of our corpus. Using the timestamp information of each tweet, all tweets created in a week were merged into a single document.

We first applied LDA to the tweets pooled by location that constituted our training data to discover topics from the online communications (Figure 1, phase 3). Within each topic, some terms have high probabilities, whereas others have low ones. After discovering the topics, the proportion of each topic for a particular week document was calculated using Bayesian inferences from the learned models (Figure 1, phase 4). To accomplish this, we used the documents in our test set that were pooled weekly based on their timestamp information. This modeling resulted in a signal indicating the variation of each topic over time. We then correlated the extracted topics with our survey items (Figure 1, phase 5). Each item measuring attitudes, knowledge, and behaviors was averaged for a particular week, and these averages were then correlated with the variation of topics over weeks.

**Table 1:** Attitudes, knowledge, and behavior questions.

Category and survey item		Survey question
**Attitude**		
	ZI-22	If there were cases of people getting infected with Zika virus in your city or town, would you approve or disapprove of special spraying at the ground level against mosquitoes to prevent the spread of the Zika virus (on a scale 1=strongly disapprove to 5=strongly approve)?
	ZI-23	If there were cases of people getting infected with the Zika virus in your city or town, would you approve or disapprove of special spraying from the air against mosquitoes to prevent the spread of the Zika virus (on a scale 1=strongly disapprove to 5=strongly approve)?
**Knowledge**		
	ZG-03b	How do scientists think someone can get the Zika virus? By sitting next to someone who has the Zika virus (on a scale 1=not likely at all to 4=very likely).
	ZG-03c	How do scientists think someone can get the Zika virus? By being bitten by a mosquito that has already bitten someone who has the Zika virus (on a scale 1=not likely at all to 4=very likely).
	ZG-05	How accurate is it to say that a pregnant woman who is infected with the Zika virus is more likely to have a baby with an unusually small head and brain (on a scale 1=not accurate at all to 4=very accurate)?
**Behavior**		
	ZG-47	If there were a vaccine that protected you from getting Zika how likely, if at all, is it that you would get the vaccine (on a scale 1=not likely at all to 4=very likely)?
	ZG-54	In the past 3 months, have you done anything to protect yourself from getting Zika (on a scale 0 to 1)?
	GM-20	In the past week, how many days, if any, did you discuss the effects of the Zika virus with family or friends (on a scale 0 to 7)?

**Figure 2:**
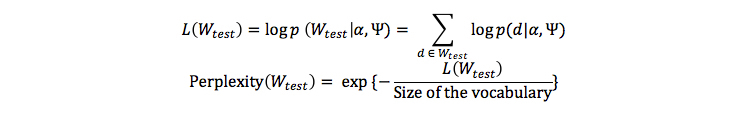
The perplexity formula used to compare the probability models. The log-likelihood of a set of held-out documents can be calculated and used for comparing the models.

LDA uses the bag-of-words approach and represents each document using a vector with the dimension of the considered vocabulary size. To examine the impact of various weighting schemes on the topic models, we compared 3 popular weighting schemes: binary, term occurrence, and tfidf representations. Topic models are probability models for a collection of documents. One approach to evaluate probability models involves comparing how well they model a held-out test set. A trained topic model is described by topic matrix Ψ and hyper parameter α for topic distribution of documents. Given those parameters, the log-likelihood of a set of held-out documents can be calculated and used for comparing the models. Traditionally, the perplexity of a model can be calculated as illustrated (see Figure 2), which shows a decreasing function of the log-likelihood of the held-out test set—the lower the perplexity, the better the model.

## Results

### Identification of Optimal Modeling Parameters

**Figure 3:**
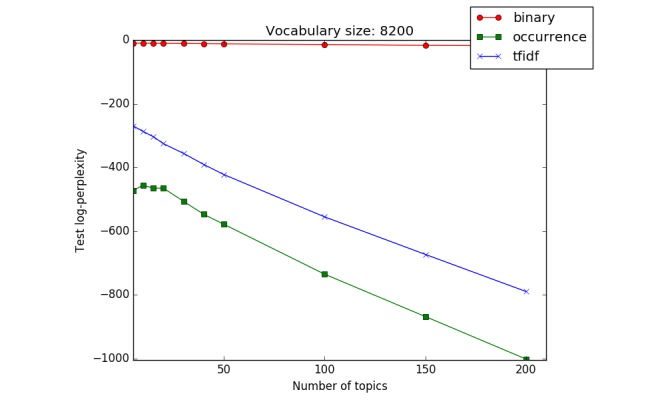
Comparison of weighting schemes (binary, term occurrence, and term frequency–inverse document frequency [tfidf]) for a vocabulary size of 8200. Perplexity of the held-out test set is the lowest for the term occurrence weighting scheme.

For different numbers of topics (k=5, 10, 15, 20, 30, 40, 50, 100, 150, and 200) and the 3 different weighting schemes (binary, term occurrence, and tfidf), we trained topic models and calculated the perplexity of the held-out test set using Bayesian inference. As mentioned before, perplexity is a common measure used to compare different probability models. The lower the perplexity on the test set, the better the model. As Figure 3 shows, the perplexity of the trained models with term occurrence weighting scheme is lower than the binary and tfidf weighting schemes. Thus, we decided to use term occurrence representation as the weighting scheme for the rest of the study.

### Probabilistic Topic Discovery

To qualitatively demonstrate the discovered topics using different topic models with k=100, 150, and 200, Table 2 represents each topic with its top 10 most probable terms. The topics in Table 2 reveal that many of the Zika-related issues such as “mosquito,” “pregnancy,” “microcephaly,” “dengue,” “Congress act for Zika funding,” “insect repellent,” “Florida,” “Miami,” “Brazil,” and “Puerto Rico” are discoverable through analysis of the content of the communications using topic modeling.

**Table 2:** Top 10 words of some of the topics of the trained latent Dirichlet allocation (LDA) models used to examine the association with the survey items. Terms that could be used to label a topic are italicized.

Topic number	Top 10 terms
4-LDA100	*zika*, *virus*, *mosquito*, health, amp, zika, *pregnant*, new, zikavirus, first
57-LDA100	*mosquito*, many, *zika*, amp, *virus*, wish, *summer*, full, look, leg
63-LDA100	*congress*, *funding*, *act*, *emergency*, tell, via2026, *approve*, add, lirikoph, zika2014fast
96-LDA100	mosquito, virus, zika, *microcephaly*, bill, *repellent*, *mosquito*, *summer*, amp, natural
2-LDA150	virus, zika, mosquito, *birth*, like, first, *health*, buy, *bill*, *dengue*
12-LDA150	virus, cdcgov, zika, via, test, *pregnant*, mosquito, *cdc*, *prevention*, amp, primarily, *contraception*
15-LDA150	virus, *zika*, mosquito, thing, hot, *congress*, pregnant, amp, *funding*, *emergency*
73-LDA150	zika, virus, *tech*, *blood*, mosquito, outbreak, *threat*, *pregnancy*, *government*, brazil
120-LDA150	zika, virus, mosquito, *zika*, health, amp, *funding*, *congress*, via, *pregnant*
149-LDA150	virus, *zika*, mosquito, around, like, *protect*, money, suspected, *health*, *travel*
10-LDA200	zika, virus, amp, *zika*, *funding*, *mosquito*, *congress*, new, like, house
37-LDA200	zika, virus, *insect*, *abortion*, virus, *repellent*, *prevent*, rubio, mosquito, pregnant
39-LDA200	mcilroy, rory, zika, virus, *pesticide*, crisis, rio, *florida*, *zika*, *vaccine*
87-LDA200	mosquito, virus, *zika*, *miami*, state, *dengue*, national, need, *fever*, month
104-LDA200	zika, virus, *medical*, *rio*, *brazil*, new, everything, *mosquito*, *baby*, amp
135-LDA200	virus, zika, *mosquito*, *central*, light, *zika*, *health*, clean, video, amp
165-LDA200	*virus*, zika, mosquito, *cdc*, breaking, *zika*, *pregnant*, amp, u.s., rather
197-LDA200	virus, zika, *rico*, *puerto*, *congress*, *funding*, obamacare, zika, emergency, scott

### Associations of Topics With Weekly Measures of Attitudes, Knowledge, and Behaviors

The associations of topics with the weekly averages of attitudes, knowledge, and behavior items appear in Table 1. As one can surmise, the content of the online communications of each week focuses on a handful of the discovered topics. In other words, there are topics that only appear in some weeks and there are a limited number of topics that appear in many. For the correlation analysis presented here, the topics that appeared in just a few (2 to 3) weeks were discarded; we only considered those topics that appeared in almost all weeks. We calculated correlations using our trained topic models with a vocabulary size of 8200, term occurrence weighting scheme, and with k=100, 150, and 200 topics. We were interested in discovering topics whose variations over time mimic the variations of the Zika survey items. Therefore, we calculated the posterior probability of all topics in each week and looked into the variation of the topics over time (weeks).

**Figure 4:**
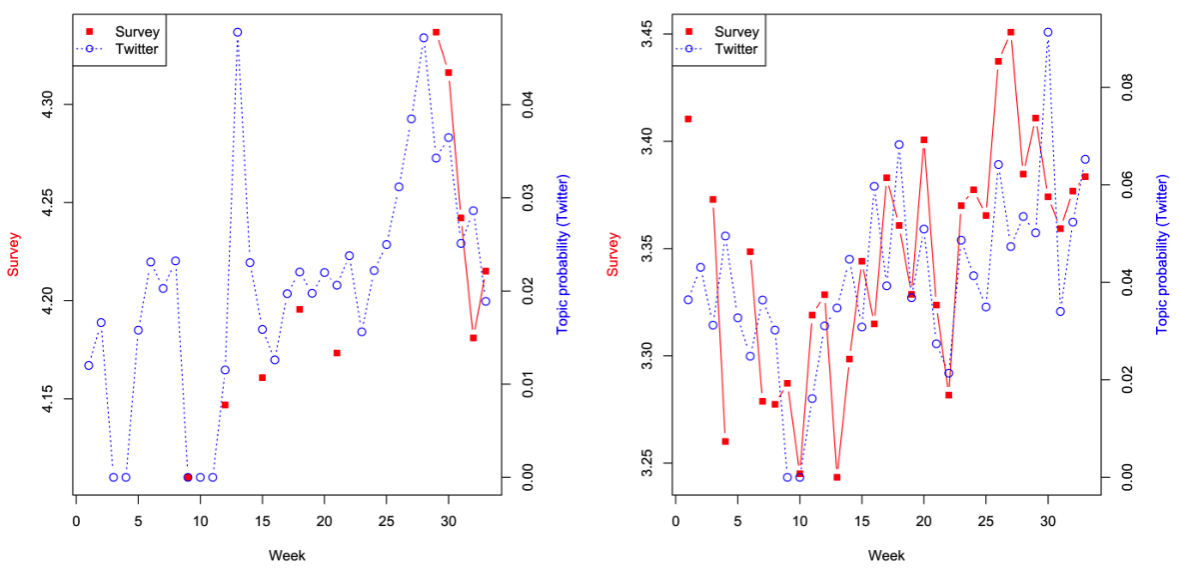
Probability of topics (circle markers) and survey items (square markers) over time. Using the trained model, the probability of each topic can be calculated in each week. The survey items at each week are the average of the participants' responses. Survey items missing in some weeks were not asked of the respondents in those weeks. Left: Attitude toward ground spraying (survey) compared with congress funding (Twitter) (197/LDA200). Right: Knowledge about microcephaly (survey) compared with Zika protection and travel (Twitter) (149/LDA150). LDA: latent Dirichlet allocation.

Figure 4 shows the variations of topics and survey items over time. Because some of the survey items were not asked of the respondents in all of the weeks, there are missing data in the survey. Since the variation of topics over time generated from our analyses can be used to monitor/predict the variation of survey measures, Twitter data can be used as a good measure to gauge attitudes, knowledge, and behaviors.

**Figure 5:**
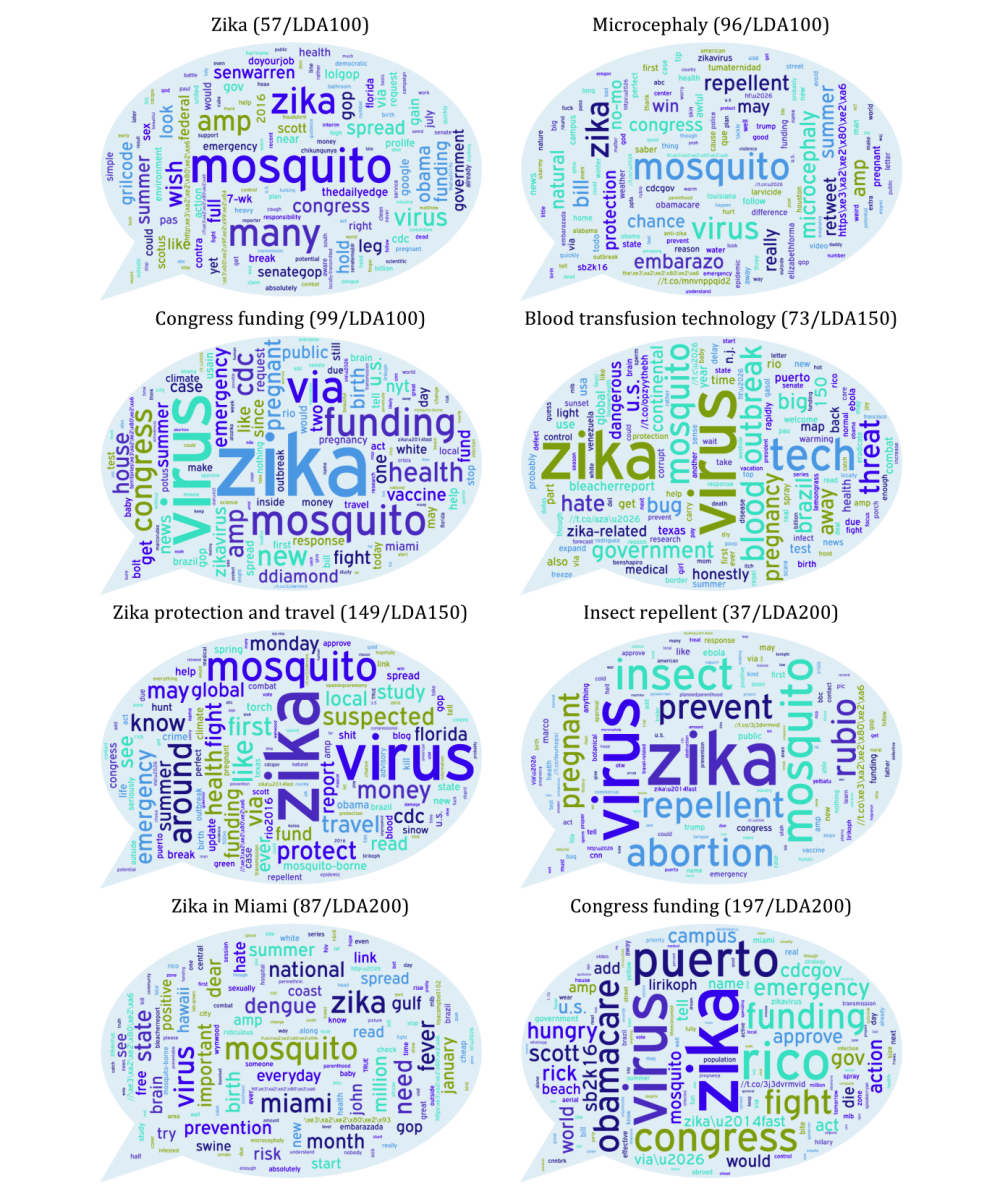
Word cloud of topics that showed significant correlation with survey items. LDA: latent Dirichlet allocation.

Table 3 shows topics with significant correlation with attitude, knowledge, or behavior questions, and Figure 5 depicts these topics with their word clouds. We have reported the *P* values for the *t* tests of the Pearson correlations to determine statistically significant correlations in Table 3.

Items ZI-22 and ZI-23 of the Zika survey measured attitudes toward ground and air spraying against mosquitoes; these correlated positively with topics about “Congress funding,” “Zika protection and travel,” and “Zika in Miami” (Table 3). “Congress funding” and “Zika protection and travel” also correlated with the microcephaly knowledge item, and this item also positively correlated with a topic that specifically captures discussions about “microcephaly” online.

According to the results (Table 3), the behavioral item GM-20 that is measuring the amount of Zika-related discussion with family/friends of the respondents correlates with “Zika,” “Zika protection and travel,” “Congress funding,” and “insect repellent” topics. Thus, our content analyses of the online communications not only discovered topics that can serve as a gauge of the amount of Zika-related discussion but also revealed the most probable topics that construct the body of their communications (in this case, “travel plan/change” and “insect repellent”). The other behavioral item of the survey that is asking about recent preventive action to avoid Zika infection correlates with the topic “insect repellent.” Some of the discovered topics do not correlate significantly with any of the Zika items. For instance, topic 104/LDA200 can be interpreted as capturing those communications regarding the “rio2016 olympics.” Because none of the analyzed survey items were directly related to this topic, there is not a significant correlation between this topic and any of the survey items. This observation indicates the possibility of discovering topics by analyzing the content of the communications that may not be measured by conducted surveys but were captured by our model.

**Table 3:** Summary of topic correlates for survey items. LDA: latent Dirichlet allocation.

Category, survey item, and topic			Correlation (*P* value)
**Attitude**			
	**Ground spraying (ZI-22)**		
		“Congress funding” (197/LDA200)	.88 (<.001)
		“Zika protection and travel” (149/LDA150)	.68 (<.001)
	**Aerial spraying (ZI-23)**		
		“Congress funding” (197/LDA200)	.92 (<.001)
		“Zika in Miami” (87/LDA200)	.67 (<.001)
**Knowledge**			
	**Microcephaly (ZG-05)**		
		“Zika protection and travel” (149/LDA150)	.52 (<.001)
		“Congress funding” (99/LDA100)	.51 (<.001)
		“Microcephaly” (96/LDA100)	.43 (<.001)
**Behavior**			
	**Getting Zika vaccine (ZG-47)**		
		“Blood transfusion tech” (73/LDA150)	–.68 (<.001)
	**Practicing any preventive behavior to avoid Zika (ZG-54)**		
		“Zika” (57/LDA100)	.65 (<.001)
		“Insect repellent” (37/LDA200)	.59 (<.001)
	**Discussing Zika with family/friends (GM-20)**		
		“Zika” (57/LDA100)	.47 (<.001)
		“Insect repellent” (37/LDA200)	.45 (<.001)
		“Congress funding” (197/LDA200)	.44 (<.001)
		“Zika protection and travel” (149/LDA150)	.30 (<.001)

## Discussion

### Principal Findings

In this paper, we used topic modeling to analyze the content of online communications on the Twitter microblogging service. Instead of relying simply on the volume of the online communications, we analyzed their content to identify topics whose variations over time could be associated with the variations in attitudes, knowledge, and behaviors measured with survey methods. After collecting a corpus of tweets related to Zika virus, we aggregated them into longer documents by either using location or timestamp information. To parse out topics of discussion, we used LDA probabilistic topic modeling and calculated the variation of topics over time using Bayesian inference. Our results demonstrated the possibility of discovering evidence from social media that enables us to identify community attitudes, knowledge, and behaviors in a timely manner at low cost. Our methodology can be applied to collections of tweets from other domains of interest, from business to politics to other public health areas.

We went beyond the frequency-based measures by analyzing the content of online discussions of Twitter. Tweets are in-the-moment updates and contain useful observations about the larger world. Analyzing the actual content of Twitter messages provides a finer granularity and enables us to identify topics of the discussions and associate particular topics to particular measures of interest. For instance, our analysis revealed that community members not only have discussed Zika with their family and friends but also that their discussions were primarily about insect repellent, Congress funding, and Zika-related travel.

Topic modeling techniques can discover patterns from a collection of text documents and automatically extract topics in the form of multinomial distributions over words. A challenge in applying topic models to any text mining problem is labeling and interpreting a multinomial topic model accurately. Interpreting the topics or labeling them is a step that is usually done manually after topic discovery. In our analyses, the correlations with attitude, knowledge, and behavior items helped to avoid the challenge of topic interpretation.

### Limitations

One of the limitations of our approach is that we are unable to discover on Twitter all of the constructs that are being measured in a survey because not all of the items of interest may appear in online communications content. However, this approach allows researchers to identify topics that have not been measured in a survey but have appeared in online discussions. These topics can then directly be measured using the proposed methodology or can be included in future surveys.

### Conclusions

In this paper, we investigated the associations between the online communications on Twitter and attitudes, knowledge, and behaviors regarding the public health challenge of the Zika virus. Our results demonstrated that it is possible to uncover topics of discussions from Twitter communications that are associated with Zika-related attitudes, knowledge, and behaviors of populations over time. Our analyses showed that the discovered topics of US congressional funding for Zika, microcephaly, Zika-related travel discussions, insect repellent, blood transfusion technology, and Zika in Miami can be used to monitor and predict the population’s attitudes toward ground and aerial special spraying, knowledge about microcephaly, and various preventive behaviors such as travel change or getting a Zika vaccine. Our work demonstrated that social media data can be used as a complementary source of information alongside traditional data sources to gauge patterns of attitudes, knowledge, and behaviors of a population and further developing and improving text mining tools have practical applications in public health domain.

## Appendix

Multimedia Appendix 1 Nationally representative telephone samples: SSRS omnibus and custom studies.

## Abbreviations

LDA: latent Dirichlet allocation
tfidf: term frequency–inverse document frequency
