# Supplementary material for: Associations of Topics of Discussion on Twitter With Survey Measures of Attitudes, Knowledge, and Behaviors Related to Zika: Probabilistic Study in the United States
Source: JMIR Public Health Surveill. 2018 Feb 9;4(1):e16. doi: 10.2196/publichealth.8186 (PMC5889815; doi:10.2196/publichealth.8186)
Supplement: Multimedia Appendix 1 [file publichealth_v4i1e16_app1.pdf]

Associations of Topics of Discussion on Twitter with Survey Measures of Attitudes, Knowledge, and Behaviors Related to Zika: A Study in the United States

Mohsen Farhadloo, PhD, University of Illinois, Urbana-Champaign, IL, USA, and Annenberg Public Policy Center, University of Pennsylvania, mfarhad@illinois.edu

Kenn Winneg, PhD, Annenberg Public Policy Center, University of Pennsylvania, PA, USA, ken.winneg@appc.upenn.edu

Man-pui Chan, PhD, University of Illinois, Urbana-Champaign, IL, USA, sallycmp@illinois.edu

Kathleen Hall Jamieson, PhD, Annenberg Public Policy Center, University of Pennsylvania, PA, USA, Kathleen.jamieson@appc.upenn.edu

Dolores Albarracin, PhD, University of Illinois, Urbana-Champaign, IL, USA, dalbarra@illinois.edu

"Corresponding Author:" Mohsen Farhadloo, PhD, University of Illinois, Urbana-Champaign, IL, USA, and Annenberg Public Policy Center, University of Pennsylvania, mfarhad@illinois.edu, 209-761-5350

Supplementary material 1

Nationally Representative Telephone Samples – SSRS Omnibus and Custom Studies

1. Coverage: To ensure representativeness, all working cell phone and landline telephone exchanges in the fifty states and the District of Columbia are covered in SSRS's overlapping, dual-frame (cell phone and landline) design. According to the most recent National Health Interview Survey (NHIS), nearly 97% of U.S. adults are reachable by either a cell phone or a landline[1].

2. Covering the cell-phone only population: Currently, nearly half of U.S. adults live in households without a landline connection. To ensure adequate representation of the cell-phone only (CPO), the following two steps are taken:
   (1) The majority of interviews are completed with respondents reached through their cell phones. On the SSRS Omnibus, the current share of respondents interviewed on their cell phones is 60%. Of these, about 60% are CPO. In other words, about 36% of

[1] https://www.cdc.gov/nchs/data/nhis/earlyrelease/wireless201612.pdf

respondents are CPO. On custom studies, the share of respondents reached via cell phone is typically 70% (approximately 40% CPO).

(2) Weighting by phone usage: Phone status, that is CPO, landline only or dual-user, is included in the post stratification weighting adjustments, based on the most recent NHIS estimates. Currently, this means that in a weighted national sample, about 50% of the sample is CPO.

3. Spanish interviewing: The Hispanic population is the most rapidly-growing ethnic group in the U.S. According to the Census, about one third of the Hispanic population are estimated to speak English less than very well, including some defined as linguistically isolated. To ensure that non-English speaking Hispanics are represented in the sample, about 3%-3.5% of interviews conducted in national surveys are completed in Spanish.

4. Probability-based sampling: To ensure unbiased sampling, both the landline and cell phone sample are generated randomly, so that phone numbers have an equal and known probability of selection (EPSEM). Furthermore, telephone exchanges are stratified by geography, to improve geographic representativeness and pulled in replicates of 100, to reduce sample variance.
    - When reaching a household by dialing a landline number a single respondent is selected through the following selection process: First, interviewers ask to speak with the youngest adult male/female at home. The term “male” appears first for a random half of the cases and “female” for the other randomly selected half. If there are no men/women at home during that time, interviewers ask to speak with the youngest female/male at home.

5. Adjustment for probability of selection: As part of the weighting process, each case is assigned a sample-weight (or baseweight) equal to the inverse of the respondent’s probability of selection. Based on Buskirk and Best (2012)[2], probability of selection is based on respondents’ probability of being selected into the landline sample and their probability of selection into the cell phone model:

$$P_{select}=P_{cell}+P_{LL}-P_{cell}*P_{LL}$$

Where P_{select} is probability of selection, P_{cell} is probability of selection into the cell phone frame and P_{LL} is probability of selection into the landline frame.

P_{cell}, in turn is equal to: $F_{cell}*N_{cell}$ and $P_{LL}$is equal to $F_{LL}*N_{LL}/Adults_{HH}$

Where F_{cell} is equal to the number of cell phone numbers selected into the study’s sample divided by the total possible cell phone numbers available for sampling, N_{cell} equals the number of cell phones by which a respondent could personally be reached, F_{LL} is equal to the number of landline phone numbers selected into the study’s sample divided by the total possible landline numbers available for sampling, N_{LL} equals the number of landlines by which a respondent’s household could be reached, and $Adults_{HH}$ is equal to the number of adults living in the respondent’s household who could be selected to be interviewed.

[2] http://www.princetonsurvey.com/filesave/304351_72969BuskirkBest.pdf

The sample-weight is calculated as:

$$1/\,P_{select}$$

6. Post-stratification adjustment: The sample weight renders the sample equivalent to a simple random sample. With this weight applied, the sample is weighted to reflect the overall makeup of the known U.S. adult population, based on known population parameters. Using the most recent March supplement of the U.S. Census Bureau's Current Population Survey (CPS), population parameters are calculated for:

   - Age (18-29; 30-49; 50-64; 65 or more) by gender
   - Race/Ethnicity: Hispanic and born in the continental U.S., Hispanic and born outside of the U.S. or in Puerto Rico, non-Hispanic White; non-Hispanic Black; non-Hispanic other.
   - Educational attainment (less than high school graduate; high-school graduate, including non-college technical degrees; some college education, including Associate's Degree; Bachelor's degree or more)
   - Census Region (Northeast; Midwest; South; West)

   In addition, the data are weighted to reflect the distribution of the population along quintiles of population density. All counties in the U.S. are ranked from least dense to most dense and assigned to ranked quintiles of about equal size, based on the most recent Decennial Census. Weighting the sample to population density improves representativeness of the weighted sample by urban, suburban and rural status.

   Post-stratification also includes the phone status variable, mentioned above, based on the most recent NHIS estimate.

   Weighting is done by iterative proportional fitting, or 'raking', a method is which the data are repeatedly weighted to the parameters until the variance between the weighted sample and the population parameters is zero, or near-zero. [3]

7. Response rate calculation: Response rate is calculated using AAPOR's response rate 3 (RR3)[4]. RR3 is calculated as the number of completed interviews (I) divided by the estimated number of eligible respondents (E). The estimated number of eligible respondents is calculated as:

   $$E=(I+P)+(R+NC+O)+e(U_{HH}+U_{O})$$

   P is partial interviews, R is eligible refusals, NC is eligible non-contacts (where a respondent was identified but no interview completed), O is other eligible cases not completed, U_{HH} are cases where a household was reached but the eligibility of respondents not ascertained, and U_{O} are other unknown cases where it is unclear whether the number is attached to a household (or cell phone respondent) and whether that respondent is eligible or not.

[3] Following post-stratification, the weights are truncated, or trimmed, at a range of 0.25 and 4 to reduce the impact of any particular case, and to control the increased variance caused by weighting.

[4] https://www.aapor.org/AAPOR_Main/media/publications/Standard-Definitions2015_8theditionwithchanges_April2015_logo.pdf

e is an estimator for the percent of unknown cases estimated to be eligible. In dual frame studies to different e estimators are used for landline and cell phone numbers:

e1 - Estimated Percentage of Screener Eligibility (i.e., the proportion of households known to be eligible at the household-level that are estimated to have an eligible respondent residing there); and e2 = Estimated Percentage of Household Eligibility (i.e., the proportion of cases that are of unknown eligibility at the household-level and it is unknown if an eligible respondent resides there).
